# Ovarian Physiology

**Published:** 1874-11

**Authors:** 


Retrograde Metamorphosis of the Graafian Follicle.—The following results are summed up by Slavjansky, as obtained from his anatomical and physiological researches:

1. The graafian follicles develop themselves from the primordial follicles, and are growing towards maturity from the first month of birth to the fortieth year.

2. The larger number of follicles do not mature, do not rupture, do not discharge their contents, but pass over into a condition of atrophy, which is analogous to the formation of the corpora lutea.

3. The development and ripening of the graafian follicle does not take place periodically in a regular manner, and there is no connection between it and menstruation.

4. Menstruation is a physiological phenomenon, unconnected with the development and ripening of the graafian follicle.

5. The rupture of the more or less ripe follicle is associated with congestions of the genital organs, which are as yet unexplained matters.

6. Certain diseases, such as fevers, poisoning, etc., cause atrophy of the follicles where there has previously been a parenchymatous inflammation of the ovary.—*Allg. Med, Central Ztz.,*

